# Non-Invasive Assessment of Physiological Stress in Captive Asian Elephants

**DOI:** 10.3390/ani9080553

**Published:** 2019-08-14

**Authors:** Vinod Kumar, Muthulingam Pradheeps, Adiseshu Kokkiligadda, Rajashekhar Niyogi, Govindhaswamy Umapathy

**Affiliations:** 1 Laboratory for the Conservation of Endangered Species, CSIR-Centre for Cellular and Molecular Biology, Uppal Road, Hyderabad 500007, India; 2 Tamil Nadu State Disaster Management Authority, Chennai 400001, India

**Keywords:** Asian elephant, non-invasive method, fecal glucocorticoid, stress, workload, management

## Abstract

**Simple Summary:**

Fecal glucocorticoid metabolite (fGCM) concentrations were examined in captive Asian elephants in relation to sex, body condition score, and working conditions. A total of 870 samples from 37 elephants in four elephant facilities were collected. We used a cortisol enzyme immunoassay for quantification of fGCM concentrations in fecal samples. Mean fGCM varied significantly across elephant facilities. Female elephants born in the wild exhibited elevated fGCM concentrations across all the facilities compared to males and captive-born elephants.

**Abstract:**

Asian elephant (*Elephas maximus*) populations, both in the wild and in captivity, have been continually declining over the decades. The present study examined the physiological stress response of captive Asian elephants in relation to body condition score and different working conditions. A total of 870 dung samples of 37 captive elephants (24 males and 13 females) from four facilities were collected to examine fecal glucocorticoid metabolite concentrations (fGCM). The elephants in forest camps with exposure to natural habitats had a higher body condition score than those in more confined spaces. Wild born elephants and females (except in one case) had higher concentrations of fGCM than captive born elephants and males, respectively. Elephants engaged in the Dussehra festival had elevated fGCM concentrations than their counterparts at Mysore zoo. We recommend a few management practices for the long-term survival of rapidly declining captive elephant populations.

## 1. Introduction

The Asian elephant, an endangered species, occurs in south and south-east Asia, with a population estimated between 40,000 and 50,000 individuals [1]. Elephant populations have continued to decline for centuries in most of their range, especially in south-east Asia, due to loss, degradation, and fragmentation of their habitat, and poaching [2,3]. However, the south Indian populations in the Western Ghats are increasing in some areas due to effective conservation measures [4]. Although elephants are an integral part of culture and civilization, still hundreds of elephants are being killed every year due to conflicts with humans. About 14,000 to 16,000 (22% to 30%) Asian elephants live in captivity [3], and these elephants are used extensively for logging, patrolling, tourism, and religious activities. Captive populations continue to decline due to failure in reproduction, diseases, and poor husbandry practices [5,6]. Traditionally mahouts (elephant trainer, rider, and keeper) manage the captive elephants, however the skills and quality of mahouts have declined due to reduced monetary benefits which affect the welfare and management of captive elephants [7]. Further, increased use of non-traditional or unskilled and inexperienced mahouts leads to physiological and psychological stress, which in turn makes the animals violent, and these violent animals could cause human casualties [7,8]. In the south Indian state of Kerala, which has a long tradition of keeping captive elephants, 274 cases of manslaughter by captive elephants have been recorded between 1989 and 2003 (an average of 10 manslaughters/year), due primarily to physical abuse, stress, and mismanagement of the elephants [9].

Although most of the captive elephants in India are managed by mahouts and veterinarians, their biological needs, welfare, and monitoring of health are not prioritized. Although the Government of India has issued standard guidelines for care and management of captive elephants, the conditions of the facilities, welfare schemes, and other husbandry practices vary widely. Interestingly, elephants in most of the state-owned camps in the forest areas have access to natural food, water, mating with wild animals, and social interaction with other individuals. However, in some elephant facilities, including the zoos, elephants do not have the same natural environment and resources, resulting in changes in their behaviors. Varma et al. [10] reported that 40% of captive elephants in India have shown aggressive or unpredictable behavior, and 13% of these elephants exhibited stereotypic behavior. Further, the report revealed that about 55% of captive elephants were involved in incidences of injuring or killing people [10]. Many studies have examined the welfare of captive Asian elephants with reference to social life [7], management factors [11,12], tourism [13], lipid profiles with reference to body conditions and adrenal glucocorticoid activity [14], stress and body condition with climate and demography [15]. In India, no detailed information is available on the physiological responses of captive Asian elephants with reference to various husbandry practices and working conditions except one study in three Indian zoos [6]. We hypothesize that poor welfare conditions and husbandry practices including different working conditions have a direct impact on the physiological status of captive Asian elephants, especially resulting in an increase in stress hormones.

Glucocorticoids (cortisol and corticosterone), the stress hormone, are released in the system to increase fitness through energy generation for the short-term, however a prolonged higher concentration of stress hormones is known to affect reproduction, immunity, and growth [16,17]. Measurement of glucocorticoids from blood [18,19] and saliva [20,21] often involves restraining or handling of animals which itself might cause stress to the animal and may confound the picture of the underlying physiology. Measurement of glucocorticoid metabolites from feces could provide an alternative to invasive measures and allow a “pooled” assessment of the adrenal activity over a period. It also minimizes the effect of pulsatile and diurnal variations of many circulating hormones [22] and it can be used for long term investigation of physiological stress in elephants [6]. A few studies have already demonstrated that physiological stress could be measured by glucocorticoid metabolites in feces across a wide range of mammals, including Asian elephants [6,23,24,25,26].

With this background, we aimed to examine fecal glucocorticoid metabolite concentrations (fGCM) concentrations in captive Asian elephants of Mysore zoo, Mysore Dussehra temporary elephant camp, Mudumalai Tiger reserve elephant camp, and Bandhavgarh Tiger reserve elephant camp.

## 2. Materials and Methods

### 2.1. Elephant Facilities

*Mysore zoo*: Elephants are maintained primarily for public display. The age of the elephants ranged from 9 to 58 years (Table 1). Males and females were kept together in a large enclosure from 8.00 h to 17.00 h unchained and the rest of the time they were individually chained in a shed.

*Mysore Dussehra elephant camp*: This is a temporary facility erected every year near Mysore palace during the Hindu festival of Dussehra. Elephants are brought to Mysore city from 4 different elephant camps of Karnataka state forests 20 days before the Dussehra festival. In this camp, elephants are chained most of the time and are taken out for the procession in the city for rehearsal daily (07:00 to 9:00 h and 17:00 to 19:00 h) till the festival ends. The elephants are sent back to their respective camps once the Dussehra festival ends.

*Mudumalai elephant camp*: This is a permanent facility managed by Tamilnadu State Forest Department and is used for tourism and patrolling. The elephants in the camp are maintained as mixed groups of adult and sub-adult males, females and calves of different age groups. These elephants are maintained under semi-captive conditions. Elephants are brought to the maintenance camps typically once or twice a day, early in the morning (06:00–08:00) and in the afternoon (16:00–18:00) for supplementary feeding, veterinary inspection, and are exhibited for the tourists. The female elephants in the camps have access to water, forest for grazing, and mating with wild and captive males.

*Bandhavgarh Tiger Reserve elephant camp*: Elephants in Bandhavgarh were brought to the national park in 1971 by Madhya Pradesh state government and are kept under semi-captive conditions in a camp. During the daytime, they are subjected to patrolling and other works inside the park. However, they are left to freely range at night for grazing.

Although there were reports of breeding of elephants in these facilities, no females were pregnant during the study period. All the individuals were healthy and no medical issues were observed. Elephants in these facilities were kept with natural flooring.

### 2.2. Sample Collection

A total of 870 fresh dung samples were collected from 37 elephants (24 male and 13 female) from Mysore zoo (*n* = 167), Mysore Dussehra elephant camp (*n* = 271), Mudumalai (*n* = 190), and Bandhavgarh (*n* = 242) tiger reserve elephant camps. Dung samples from Mysore Zoo and Mysore Dussehra elephants were collected daily between September and October 2014. In Bandhavgarh, the samples were collected on alternative days between January and May 2014, while samples in Mudumalai were collected from January to August 2013. Details of elephant age/sex, number of samples collected, body conditions, and other information are given in Table 1. Dung samples were collected between 8:00 to 9:00 h. Since no laboratory or freezer (−20 °C) was available in most of the locations, freshly collected samples were air dried the same day using an oven at 60 °C–80 °C, pulverised, and stored in sealed containers with silica beads at 4 °C. These samples were shifted to the freezer (−20 °C) within a week and stored until further analysis [28].

### 2.3. Body Condition Score (BCS) and Other Information

The Body Condition Score (BCS) was estimated numerically between 0–11 based on the condition of six different regions (head, scapula, thoracic region, flank area, lumbar vertebrae, and pelvic bone) of the body as reported previously [27]. Data on musth condition and major injury, if any, were also recorded during sample collection. Information on captive born or wild caught, as well as the previous history of killing humans, were also collected from respective forest department records, previously published information, and online databases [29,30].

### 2.4. Physical Activities

Elephants in Mudumalai and Bandhavgarh are regularly used for rides for tourists and patrolling activities in the forests. The elephant ride consists of a 4–5 km ride in the forests carrying 4 to 6 people on their back for about 2–3 h both in the morning and the evening hours. Some elephants are subjected to do patrolling inside the park for about 4–5 h, mostly carrying two persons on their back. Elephants in the Dussehra facility were taken out for rehearsal, which covered two walks with a distance of 3 km (07:00 to 09:00 a.m. and 05:00 to 07:00 p.m.) around the palace and through the town every day for 10 days. During rehearsal, the elephants would also carry weight, which varied from 100 kg to 350 kg. After rehearsal, elephants were chained and allowed to rest in their respective confinement. On the final day of the festival, 12 elephants participated in the procession from Mysore palace to Bannimantap (5 km), along with traditional armies of Mysore Maharaja with royal respects marking the end of Dussehra. Mysore zoo elephants were not involved in any physical activities during our study period.

### 2.5. Extraction of Steroid Metabolites

The dung samples were extracted according to a previously described procedure [6,23]. The pulverized dung sample was sieved, and 0.2 g of fecal powder was transferred into a 15 mL falcon tube, 5 mL of 80% methanol was added, and it was vortexed vigorously for 20 min. Then, the sample was centrifuged at 3300× *g* for 20 min; the supernatant was transferred into 5 mL of cryogenic vial and stored at −30 °C until further assay. Extraction efficiency was calculated by adding a known amount of ^3^H labeled cortisol in fecal samples before extraction [6,31]. Extraction efficiency was found to be 85.6% ± 6.3 for cortisol.

### 2.6. Glucocorticoid Immunoassay and Procedure

fGCMs were measured using a single antibody competitive enzyme immunoassay incorporating a polyclonal cortisol antibody (R4866), as described earlier [6,32]. The cortisol antibody was diluted to 1:9000, HRP (Horseradish peroxidase)-conjugated cortisol 1:250,000 (Dr. C. Munro, University of California, Davis, CA, USA) and cortisol standards used as 1000–1.9 pg/well. The cortisol assay was successfully validated in Asian elephants for reliable quantification of adrenal activity [6].

Cortisol antibody was diluted in coating buffer (0.05 M sodium bicarbonate buffer, pH 9.6) before coating a 96-well plate (Nunc-Immuno maxisorp, Thermo Fisher Scientific, Roskilde, Denmark), and being incubated overnight at 4 °C and washed four times with washing buffer (0.15 M NaCl and 0.05% Tween 20, Sigma, India). Fecal extracts (50 µL) or cortisol standards diluted in assay buffer (0.1 M PBS, pH 7, containing 0.1% BSA) were added to the wells immediately followed by 50 µL of HRP (Horseradish peroxidase) conjugated cortisol. After 2 h of incubation at room temperature, the plate was washed and 50 µL of TMB/H_2_O_2_ (Genei, Bangalore, India) substrate added and kept in the dark for 10–15 min. The reaction was stopped by the addition of 50 µL of 1N HCL and absorbance read at 450 nm in the ELISA reader (Thermo Multiskan Spectrum Plate Reader, version 2.4.2, Thermo Scientific, Vantaa, Finland).

Parallel displacement curves were made between pooled serial dilution of fecal extract (endogenous antigen) and respective standards (exogenous antigen) [6]. The cortisol antibody sensitivity was calculated to be 1.95 pg/well at 90% binding. The recovery of known amounts of unlabeled steroid was 87.3 ± 2.3%. The correlation (r^2^) and slope (m) value for the recovery of standards were r^2^ = 0.99 and m = 0.93, respectively. Intra- and inter-assay coefficient of variation (CV) were 3.96% and 7.86% (*n =* 25 plates). The cortisol antibody cross-reacts with cortisol 100%, prednisolone 9.9%, prednisone 6.3%, cortisone 5%, and <1% with corticosterone, desoxycorticosterone, 21 desoxycortisone, testosterone, androstenedione, androsterone, and 11-deoxycortisol [33].

### 2.7. Statistical Analysis

Data are presented as mean ± S.E.M. We used the Friedman repeated measures ANOVA to test for difference of BCS among the elephant facilities. Data analysis was carried out location wise as samples were collected in different months and from different geographical locations. The Student’s *t*-test was used to examine the significant difference between two variables (sex versus BCS). We used one way analysis of variance to examine the difference between the variables. We used the Pearson correlation (r) to test the relationship between the variables (age versus body condition score; fecal glucocorticoid metabolites concentrations versus body conditions score). A single BCS was assigned to each elephant as no difference was observed during the study period. We used the general linear model (GLM) procedures with repeated measures to examine changes in hormone concentrations within and between the facilities with reference to age (in years), sex (male and female), physical activities (whether it was involved in 3 h of physical activity or not), weight type (weight carried, yes or no), weight carried (actual weight carried during rehearsal such as 100, 200, 350, and 1150 kg), working hours (involved in patrolling, procession), participation in the public procession (yes or no), chained hours, and body condition score (0–11). We used BCS and age as fixed factors to avoid pseudo-replication in the model, and further parameter estimation was carried out following the hybrid method with maximum iterations of 100. We compared fGCM data between Mysore zoo and Dussehra facilities to understand stress responses due to an elephant’s participation in public procession as these two facilities share identical husbandry practices such as food, health monitoring, weather, and season. We did not observe musth condition or any major injury to elephants during our study, so we did not include these variables in the analysis. All statistical analyses was performed using SPSS 17.1 ver. (IBM corporation, New York, NY, USA).

## 3. Results

### 3.1. Body Condition Score (BCS)

Overall, the mean body condition score of elephants was 7.0. It ranged between 4 and 8 (Table 2) and significantly varied across the facilities (GLM F_3,869_ = 40.90, *p* = 0.001) where the forest camp elephants at Mudumalai and Bandhavgarh had higher body condition scores than those in Mysore Zoo and the Dussehra facility. Body condition score was found to be negatively correlated with the age of elephants (r = −0.37, *n* = 37, *p* = 0.024) and males had significantly higher body condition scores than females across all the facilities (T = 3.69, *p* = 0.001).

### 3.2. fGCM Concentrations

**Figure 1:**
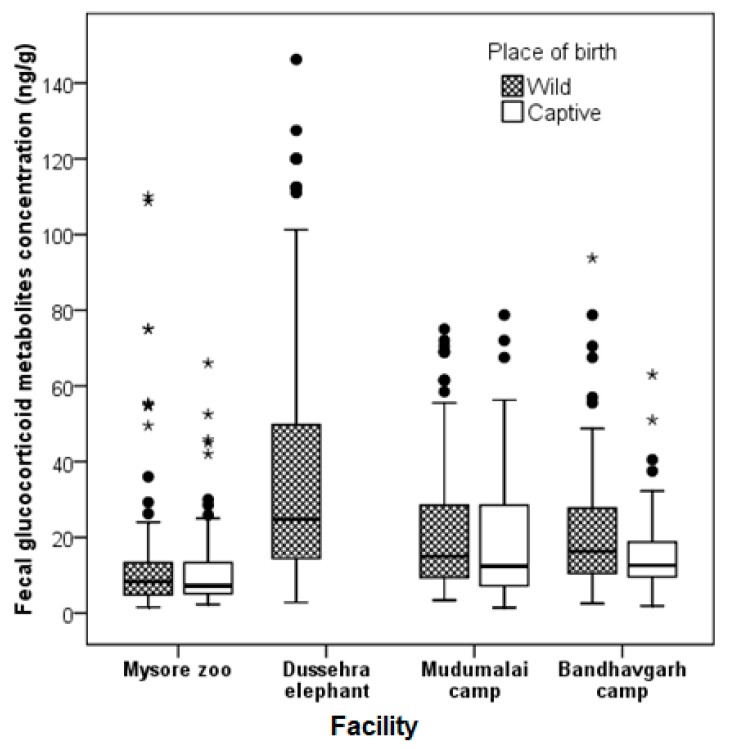
Box plot showing fGCM concentrations of elephants born in captive and the wild in four elephant facilities. In the Dusshera camp, all the elephants were wild caught (Filled rounds = outliers, filled asterisk = extreme values).

**Figure 2:**
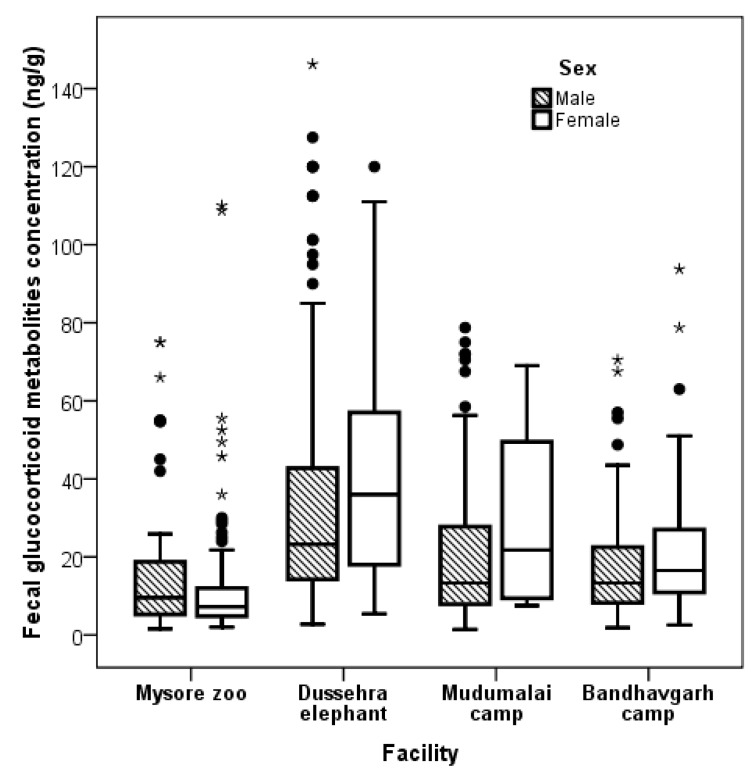
Box plot showing fGCM concentrations between male and female elephants in four elephant facilities (Filled rounds = outliers, filled asterisk = extreme values). In Mudumalai camp, the female’s value was from one individual.

Overall, fGCM concentrations did significantly differ for facilities (GLM F_3,896_ = 14.94, *p* = 0.0001), sex (GLM F_3,869_ = 9.80, *p* = 0.002), body condition (GLM F_5,869_ = 4.48, *p* = 0.003), birth location (GLM F_1,869_ = 4.89, *p* = 0.003, in Dussehra camp, all elephants were wild caught), and age (GLM F_26,869_ = 7.30, *p* = 0.001), but did not significantly differ for physical activities (GLM F_1,869_ = 1.98, *p* = 0.12) (Table 2; Figure 1 and Figure 2). Further, a weak negative correlation was observed between fGCM concentration and BCS among the facilities (r = −0.08, *p* = 0.02). A weak positive correlation was observed between fGCM concentrations and age of the elephants (r = 0.17, *p* = 0.001).

### 3.3. Mysore Zoo Elephants

The mean fGCM concentration for Mysore zoo elephants was 14.7 ± 1.3 ng/g and it ranged from 7.36 to 21.55 ng/g. Interestingly, males had significantly higher fGCM concentrations than females (GLM F_1,166_ = 105, *p* = 0.001). The body condition ranged from 4.5 to 8 and it was significantly correlated with fGCM (r = 0.24; *p* = 0.002).

### 3.4. Dussehra Procession Elephants

**Figure 3:**
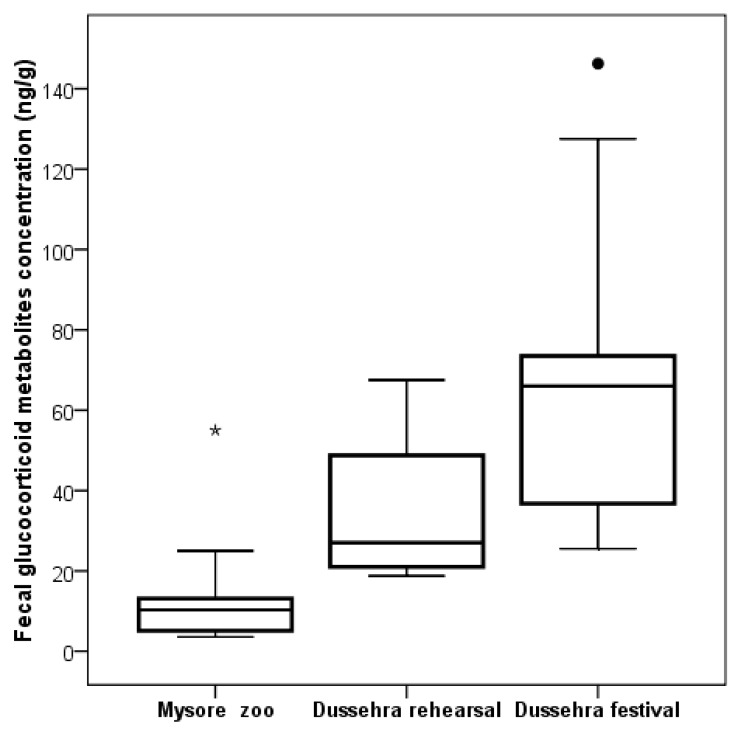
fGCM concentration of elephants from Mysore zoo, Dusshera rehearsal, and the Dussehera festival day. fGCM values in facilities were significantly different (*p =* 0.01). (Filled rounds *=* outliers, filled asterisk *=* extreme values).

A total of 271 samples from 14 elephants were collected for examining fGCM concentrations in the Dussehra elephant camp facility. The mean fGCM concentration of an individual elephant during the stay at the Dussehra festival ranged from 22 ± 5.9 ng/g (male, Srirama) to 56.54 ± 5.4 (female, Varalakshmi) and females had significantly higher fGCM (45.53 ± 3.3) than males (33.91 ± 2.08 ng/g; GLM F_1,270_ = 4.04, *p* = 0.045). Furthermore, the fGCM concentration significantly increased with more working hours (GLM, F_1,270_ = 5.7, *p* = 0.019), more weight carried (F_3,270_ = 3.4, *p* = 0.021), and lower BCS (GLM F_5,270_ = 2.7, *p* = 0.024). Overall, the Dussehra elephants showed a significantly higher mean fGCM concentration (36.26 ± 1.7 ng/g) compared to the Mysore zoo elephants (14.7 ± 1.3, GLM F_1,2437_ = 77.44, *p* = 0.001 Figure 2). Further, elephants who participated in the final day of public procession had significantly higher fGCM concentrations than elephants during rehearsal and from Mysore zoo (F = 11.89, *n* = 2, *p* = 0.001, Figure 3).

### 3.5. Mudumalai Elephant Camp

The mean fGCM concentrations ranged from 10.02 ± 1.46 ng/g to 29.36 ± 3.58 ng/g. A single female in this camp had higher fGCM (29.8 ± 4.2) concentration than that on average of all the males (19.65 ± 1.3). Overall, fGCM concentrations varied significantly among the individuals (GLM F_7,189_ = 3.61, *p* = = 0.001), however no significant difference was observed between the working and non-working elephants (GLM F_1,189_ = 0.61, *p* = 0.41). The latter might be due to the low sample size (*n* = 4). All animals were found to be healthy, and the body condition ratings ranged between 6.5 and 8.5.

### 3.6. Bandhavgarh Tiger Reserve Elephant Camp

In Bandhavgarh, overall mean fGCM concentration ranged from 11.96 ± 1.33 ng/g to 24.57 ± 2.64 ng/g and varied significantly among the individuals (GLM F_6,241_ = 3.09, *p* = 0.006). The lowest mean fGCM was observed in Sundaraj (male) which was usually on rest and the highest in Toofan (female) which was involved in patrolling during the study period. Overall, working elephants had higher mean fGCM concentration (20.48 ± 1.33 ng/g) than non-working elephants (17.60 ± 1.06 ng/g), although the difference was not statistically significant (GLM F_1,241_ = 2.59, *p* = 0.108).

## 4. Discussion

The present study revealed that the body condition and physical activities (working conditions) are significantly related to fGCM concentration. Furthermore, results also showed that fGCM significantly varied across the elephant facilities and showed higher fGCM in the Dussehra camp elephants than in the zoo elephants.

Although body condition of the animal is directly related to age and availability of resources in the natural environment, under captivity, it is further influenced by the husbandry practices. Body condition is an indicator of an animal’s fat reserve, which is directly related to healthiness [34]. Body condition score or index (BCS) helps to understand the nutritional status [35], climate and demography [15], reproduction [36], habitat conditions [37], and disease status [38] in a wide range of animals. BCS is part of animal health management in a wide range of animals to examine fatness. In the present study, BCS was significantly related to age and facilities. The mean BCS was higher in forest department owned elephant camps (Mudumalai and Bandhavgarh) than other facilities. Elephants in the forest department camps had unlimited access to resources during non-working hours, while in other facilities, the resources were limited and time-regulated. Furthermore, elephants in these camps are released into the forests during the resting period or nonworking hours and have access to mating with wild and other captive animals. Unlike elephants kept in zoos and temples, these elephants in forest camps experience near natural living conditions. Thus, resources availability and regular exposure to their natural environment might be the main reasons for the higher body condition scores. In this study, females had low BCS compared to male elephants in most of the facilities. This might be because many (5 out 15) of the females were quite old (≥50 years) and a few of them were reproductively active and had given birth previously. A similar observation was made among the female elephants of Bandipur and Nagarhole National parks and the reason was attributed to the seasonal changes in resources availability [37]. Mumby et al. [15] reported that body weight was significantly correlated with rainfall and fGCM concentrations in captive Asian elephants. In this study, a weak negative correlation was observed between fGCM concentration and BCS across all facilities. However, in Mysore zoo, a weak positive correlation was observed, which might be due to non-involvement in major physical activities, and many individuals had lower BCS. Our previous study on captive Asian elephants in three south Indian zoos showed that fGCM concentration increased with the decrease of BCS [6]. In many species, the stress level increased with declining body mass, however the impact is species-specific [39]. Since the present study animals were maintained and fed by facilities, the body condition is related to resource availability, workload, and the health status of animals. The health status of animals was found to be normal as they were periodically monitored as part of management, however the resource availability and physical activities vary widely across the facilities, which might be the main reason for the difference in the fGCM concentrations. Elephants in Mudumalai and Bandhavgarh were used for patrolling and tourist safari ride activities, which involves 3–4 working hours. During this period, the elephants are not allowed to access the resources including water; however, in the evening, they were given food supplements with regular fodder. Although marginally elevated fGCM concentrations were recorded in those elephants that were used in tourism and patrolling activities, the difference was not significant. This might be due to the small sample size and these elephants being within a natural habitat condition with sufficient resources. Unlike elephants in the forest habitats, the Dussehra procession elephants have to walk on tar roads, where many people would closely follow them during the daily familiarization and final procession. The Dussehra elephants are covered with cloths, paints, and other ornaments before they are assembled at a common place in front of the temple where people seek greetings and spend some time with them. In the evening, they are taken as part of the procession for 5 to 6 km in the busy city roads where people follow and greet them from both sides. This activity is carried out for a period of 10 days before the final day where a huge crowd of about 100,000 people gathers for the public procession. This activity lasts for 6–8 h with 3 to 4 mahouts guarding each elephant. This activity led to significant increases of fGCM concentrations from the rehearsal values in all elephants (Figure 3). Further, on the festival day, one of the elephants (Arjuna) carried the “Ambari” (mounted with Golden chariot with Goddess “Chamundeswari”), which weighs around 1.24 tonne, resulting in the increase of fGCM concentration to three times the basal level (data not shown). Throughout the year, these elephants never see or are exposed to so many people at a time and never walk on the tar roads. We also observed that a few elephants, who showed very aggressive behaviors following the initial days of processions, were given rest to avoid further untoward incidences during the festival. The increase of fGCM concentrations shows that there was a significant rise in HPA axis activation during the festival, which could indicate compromised welfare of these elephants.

A study in the African elephants has shown that individuals that interacted with humans and that were exposed to episodic loud noises had significantly elevated fGCM than those without [26]. Further, we have also previously shown that the elephants involved in the public procession had significantly increased stress levels that directly affected the female estrous cycle [6].

Overall, workload (physical stress) of elephants varied significantly with varying husbandry practices, from just patrolling a few hours in the forests to participating in the public processions with a heavy load for many hours on tar roads. This has resulted in varying responses of fGCM concentrations (Figure 3). Acute stress in elephants could cause infertility in captive elephants, of which are already suffering from limited breeding success in India. Moreover, glucocorticoid elevation in terms of the stress response can be deleterious under repeated or prolonged exposure to the stressors. This prolonged exposure leads to several stress-induced detrimental effects resulting in hyperglycemia, suppression of immune response, imperfect wound healing, and neuronal cell death [40].

The management recommendation includes minimizing participation in religious activities, processions, forest department activities, etc. Opportunities should be created for elephants to interact with other elephants in the facility, especially for females, and enough time should be provided for exercise in an open area with others. Females of reproductive age (20–55 years) should not be used for stressful activities as a prolonged elevated level of stress affects the reproductive cycle [6]. Adult male elephants may be used for tourism and patrolling activities that involve 3 to 4 h per day in the forests with adequate rest on alternative days. Periodic monitoring of health and reproductive status by outside experts is required. Finally, educating the elephant handlers regarding the welfare of elephants is of utmost importance.

## 5. Conclusions

The present study indicates that non-invasive analysis of fGCM concentrations can be used to assess physiological stress associated with various husbandry practices, age/sex, and body condition, which could be used to guide the management of captive Asian elephants.

## Figures and Tables

**Table 1:** Details of Facility, ID, body condition, age, sex, number of samples collected, and origin of captive Asian elephants.

S No	Location/Facility	Elephant Name (ID)	Sex	Age as of Oct 2014 (Years)	Body Condition Score *	No. of Samples Collected (N)	Origin
1	Mudumalai camp	Udayan	Male	16	8	28	Captive born
2	Mudumalai camp	Wilson	Male	26	8	25	Captive born
3	Mudumalai camp	Sujay	Male	41	7	27	Captive born
4	Mudumalai camp	Vijay	Male	41	8	18	Captive born
5	Mudumalai camp	John	Male	23	8	25	Wild caught
6	Mudumalai camp	Sankar	Male	45	7	21	Wild caught
7	Mudumalai camp	Ganesh	Male	45	8	33	Wild caught
8	Mudumalai camp	Senthil Vadivel	Female	43	7	34	Wild caught
9	Mysore zoo	Gajalakshmi	Female	35	4.5	21	Wild caught
10	Mysore zoo	Airavathy	Female	11	6.5	21	Captive born
11	Mysore zoo	Abhimanyu	Male	10	8	21	Captive born
12	Mysore zoo	Madesha	Male	12	8	20	Wild caught
13	Mysore zoo	Padhmavathi	Female	60	5	21	Wild caught
14	Mysore zoo	Kollegala	Female	22	8	21	Wild caught
15	Mysore zoo	Rama	Male	21	7	21	Wild caught
16	Mysore zoo	Aishwarya	Female	22	7.5	21	Wild caught
17	Dussehra camp	Abhimanyu	Male	48	6.5	23	Wild caught
18	Dussehra camp	Varalakshmi	Female	59	4	20	Wild caught
19	Dussehra camp	Gajendra	Male	59	8	18	Wild caught
20	Dussehra camp	Mary	Female	58	4.5	21	Wild caught
21	Dussehra camp	Balrama	Male	56	7.5	23	Wild caught
22	Dussehra camp	Gopalasamy	Male	32	8	23	Wild caught
23	Dussehra camp	Gopi	Male	32	8	20	Wild caught
24	Dussehra camp	Vikrama	Male	41	8	16	Wild caught
25	Dussehra camp	Arjuna	Male	54	6.5	21	Wild caught
26	Dussehra camp	SriRama	Male	56	7.5	17	Wild caught
27	Dussehra camp	Durga	Female	51	6	20	Wild caught
28	Dussehra camp	Harsha	Male	40	8	22	Wild caught
29	Dussehra camp	Kaveri	Female	36	6	12	Wild caught
30	Dussehra camp	Prashanth	Male	58	7.5	15	Wild caught
31	Bandhavgarh camp	Anarkali	Female	50	7	47	Captive born
32	Bandhavgarh camp	Sundargaj	Male	27	7	21	Captive born
33	Bandhavgarh camp	Toofan	Female	63	7	44	Wild caught
34	Bandhavgarh camp	Rama	Male	15	7	38	Wild caught
35	Bandhavgarh camp	Vanraj	Male	26	7	23	Wild caught
36	Bandhavgarh camp	Goutam	Male	68	7	16	Wild caught
37	Bandhavgarh camp	Sudarshni	Female	33	8	53	Wild caught

* Body condition rating was done using criteria described in Wemmer et al. [27].

**Table 2:** Data on sample collection, mean ± SEM body score, fecal glucocorticoid metabolite concentrations (fGCM), and management information.

Facility Name	Number of Animals	Number of Samples Collected	Mean Body Score (Mean SEM)	Mean fGCM Concentrations	Mean Working or Physical Activities (h)	Mean Chained Hours per Day (24 h)
Mysore zoo	8	167	6.81 ± 0.49	14.00 ± 1.37	00	15
Dussehra camp	14	271	6.86 ± 0.36	36.23 ± 1.78	5.94 ± 0.80	18
Mudumalai camp	8	190	7.63 ± 0.18	21.25 ± 1.29	3.73 ± 0.10	12
Bandhavgarh camp	7	242	7.14 ± 0.14	19.23 ± 0.88	3.83 ± 0.12	12
